# Mean Platelet Volume-to-Platelet Count Ratio, Mean Platelet Volume-to-Lymphocyte Ratio, and Red Blood Cell Distribution Width-Platelet Count Ratio as Markers of Inflammation in Patients with Ascending Thoracic Aortic Aneurysm

**DOI:** 10.21470/1678-9741-2019-0348

**Published:** 2020

**Authors:** Yusuf Kenan Tekin, Gülacan Tekin

**Affiliations:** 1 Department of Emergency Medicine, Sivas Cumhuriyet University, Faculty of Medicine, Sivas, Turkey.; 2 Department of Cardiology, Sivas Cumhuriyet University, Faculty of Medicine, Sivas, Turkey.

**Keywords:** Thoracic Aortic Aneurysm, Mean Platelet Volume, Platelet Count, Lymphocyte, Neutrophils, Inflammation, C-Reactive Protein, Blood Platelets

## Abstract

**Objective:**

Ascending thoracic aortic aneurysm (ATAA), seen in adults, is an important cause of morbidity and mortality. In this study, we aimed to evaluate the levels of mean platelet volume (MPV), mean platelet volume-to-platelet count ratio (MPVPCR), mean platelet volume-to-lymphocyte ratio (MPVLR), and red cell distribution width platelet count ratio (RDWPCR) in patients with thoracic aortic aneurysm.

**Methods:**

105 patients admitted to the emergency department were diagnosed with thoracic aortic aneurysm between January and December 2014, and 100 healthy individuals were involved in this retrospective study. MPV, MPVLR, MPVPCR and RDWPCRs were calculated at the time of admission.

**Results:**

Platelet and lymphocyte levels were found to be significantly lower in the patient group when compared to the healthy group (*P*<0.001, *P*<0.001, respectively), while MPV, MPVPCR, MPVLR and RDWPCR were found to be significantly higher (*P*<0.001, *P*<0.001, *P*<0.001, and *P*=0.013, respectively). In the patient group, the high-sensitivity C-reactive protein was significantly higher (*P*<0.001), and the neutrophil (*P*=0.062) was also higher. In ROC analysis, MPVPCR had the highest sensitivity (80%) and RDWPCR had the highest specificity (72%).

**Conclusion:**

The results for MPV, MPVPCR, MPVLR and RDWPCR can be evaluated as useful parameters in the emergency clinical approach in the evaluation of inflammatory activity in ATAA patients. More extensive studies are required to address the role of these parameters in determining the severity of the disease.

**Table:** 

Abbreviations, acronyms & symbols				
AA	= Aortic aneurysm		MPVPCR	= Mean platelet volume-to-platelet count ratio
ATAA	= Ascending thoracic aortic aneurysm		PLR	= Platelet-to-lymphocyte ratio
AUC	= Area under the curve		RDWPCR	= Red cell distribution width-to-platelet count ratio
COPD	= Chronic obstructive pulmonary disease		ROC	= Receiver operating characteristic
DM	= Diabetes mellitus		SD	= Standard deviation
ED	= Emergency department		SLE	= Systemic lupus erythematosus
hs-CRP	= High-sensitivity C-reactive protein		SPSS	= Statistical Package for the Social Sciences
LMR	= Lymphocyte-to-monocyte ratio		STEMI	= ST-segment elevation myocardial infarction
MPV	= Mean platelet volume		TAA	= Thoracic aortic aneurysm
MPVLR	= Mean platelet volume-to-lymphocyte ratio			

## INTRODUCTION

Aortic aneurysm (AA) is defined as the permanent expansion of more than 50% of the normal values of the transverse diameter of any segment of the aorta that should be in the range in accordance with the age and body surface of a person. Even though AA can be seen in both thoracic and abdominal aorta, it is estimated that the incidence of thoracic aortic aneurysm (TAA) has increased^[1]^. The fact that patients remain asymptomatic until the development of dissection and rupture is especially a cause of mortality and morbidity in patients in ascending thoracic aortic aneurysm (ATAA). These patients are generally diagnosed when evaluating imaging tests performed for other purposes^[2]^. The risk factors that take part in the formation and development process of the aneurysm are like the risk factors for coronary artery disease^[3]^. It is known that there are many cytokines, which cause local and systemic effects and medial degenerative changes, in the etiopathogenesis of AA. Therefore, AA is also considered an inflammatory response. In pathogenesis, in addition to lymphocytes and protease activity, the macrophages, which are accepted as the source of pro-inflammatory cytokines such as IL6, have been shown to be involved^[4]^. The majority of untreated ascending aortic aneurysms progress in a deadly manner, due to rupture or dissection^[5]^. Therefore, inflammation is important not only in the formation of cardiovascular diseases, but also in the complications that can occur subsequently^[6]^.

Recent studies have shown that mean platelet volume (MPV) can be used as a diagnostic marker for certain inflammatory disorders. MPV is a marker of activated platelets and is associated with different inflammatory conditions. In patients with diabetes mellitus (DM), cardiovascular disease, peripheral artery disease and cerebrovascular disease, increased MPV levels are associated with a low degree of inflammatory status. However, in patients with ulcerative colitis, rheumatoid arthritis and ankylosing spondylitis, high-grade inflammatory diseases, such as Mediterranean fever, are associated with decreased MPV^[7]^.

The mean platelet volume-to-platelet count ratio (MPVPCR) has been reported to be predictive of long-term mortality in many diseases, including ischemic cardiovascular diseases, sepsis, and nonalcoholic fatty liver disease^[8,9]^.

The mean platelet volume-to-lymphocyte ratio (MPVLR) may be an independent indicator for early and late mortality after ST-segment elevation myocardial infarction (STEMI), as well as predicting in-hospital mortality^[10]^.

The red cell distribution width-to-platelet count ratio (RDWPCR) is shown to be predictive of the severity of liver fibrosis in nonalcoholic fatty liver disease^[9]^.

Reducing inflammation in patients with AA can mean a reduction in existing complications. There is still a lack of biomarkers to assess the risk of aneurysm formation, enlargement or rupture. No study evaluating proinflammatory levels of MPV, MPVPCR, MPVLCR and RDWPCR in patients with ascending aortic aneurysms has been determined in the literature. In this study, we aimed to evaluate the levels of MPV, MPVPCR, MPVLR and RDWPCR in patients with thoracic aortic aneurysms.

## METHODS

Patients who were admitted to the emergency department (ED) between January 2014 and December 2014, with complaints of chest pain and respiratory distress and identified by ED diagnosis of “aortic aneurysm” and “aortic dissection”, were further analyzed to select only those with thoracic aortic aneurysm and thoracic aortic dissection. In addition, the medical records of hospitalized patients were reviewed for thoracic aortic dissection and aneurysm. In total, 105 patients were included in the retrospective study as a case group. Moreover, a total of 196 healthy individuals selected from the hospital records within the same period were involved in the study as a control group. In the study, patients previously diagnosed with hematological malignancy, chronic obstructive pulmonary disease (COPD), autoimmune liver disease, cirrhosis, metastatic bone marrow infiltration, and acute or chronic inflammatory disease such as physical trauma, tonsillitis, asthma, rheumatoid arthritis and active hepatitis were excluded from the study. Other exclusion criteria were evidence of current or recent treatment (in the past 3 months) with oral or intravenous steroids or other medications that might cause pancytopenia. Clinical, demographic and laboratory data of each patient were obtained and recorded. Then, age and gender distribution were calculated and ATAA, MPV, MPVLR, MPVPCR and RDWPCR ratios were determined. The diagnosis of ascending aortic aneurysm (40 mm≥) was confirmed by examining the patients with contrast-enhanced thorax tomography and then by an independent radiologist. Patients included in the study (study group) were compared with normal individuals with normal ascending aorta diameter, age, gender, hypertension, and DM with similar distribution (control group). This research was approved by the Human Ethics Committee (2016-03/04).

### Statistical analysis

Data were analyzed using SPSS 22.0 (SPSS Inc., Chicago, Illinois, USA). Variables were expressed as mean ± standard deviation (SD) and median (25^th^, 75^th^ percentile) as appropriate. Significance test (independent t-test) and Mann-Whitney U test were used for the comparison of research and control groups. Receiver operating characteristic (ROC) curve analysis was performed and optimal cutoff values were determined for inflammation. An alpha error of 5% was accepted.

## RESULTS

There was no statistically significant difference between the study and control groups in terms of age, gender, hypertension and DM (*P*>0.05). However, the mean aortic diameter in the case group (42.3±2.7) was significantly higher than the mean in the control group (35.8±3.2) (for both parameters, *P*<0.001) (Table 1).

**Table 1:** Demographic characteristics of the case group versus control group.

Characteristics		Case group (n=105, %)	Control group (n=196, %)	*P*-value (chi-squared test)
Age, years (mean±SD)		70.83±11.1	70.56±9.4	0.816*
ATAA (mean±SD)		42.3±2.7	35.8±3.2	<0.001*
Gender	Male	57 (54.3)	105 (53.6)	0.906
	Female	48 (45.7)	91 (46.4)	
Diabetes mellitus	Present	35 (17.9)	12 (11.4)	0.143
	Absent	161 (82.1)	93 (88.6)	
Hypertension	Present	47 (44.8)	84 (42.9)	0.751
	Absent	58 (55.2)	112 (57.1)	

* Student *t*-test

ATAA=ascending thoracic aortic aneurysm

The median values of platelet and lymphocyte count in the study group were significantly lower [201.0 (168.0-253.0) and 1.4 (1.0-1.8), respectively)] compared to the control group [254.0 (214.0-298.3) and 1.7 (1.4-2.3)], respectively), and this difference was statistically significant (*P*=0.001). Furthermore, when the two groups were compared in terms of MPV values, it was found that the value in the case group (9.7±1.1) was significantly higher than the control group (9.2±0.8) (*P*<0.001).

In the study, the mean values of MPVPCR, MPVLR, RDWPCR and high-sensitivity C-reactive protein (hs-CRP) in the case group were significantly higher than the mean values of the control group (0.05±0.02 *vs.* 0.04±0.01; 9.18±6.8 *vs.* 5.6±1.9; 0.08±0.06 *vs.* 0.06±0.02 and 14.1±6.0 *vs.* 1.6±1.06; *P*<0.001, respectively) (*P*=0.013 for RDWPCR only) (Table 2).

**Table 2:** Mean and median values of blood parameters of the case group *versus* control group.

Variables	Case group (n=105, mean±SD)	Control group (n=196, mean±SD)	Student *t*-test
White blood cell* (10^3^/µl)	6.8 (5.4-8.7)	7.2 (4.6-11.2)	0.414**
Hemoglobin (g/dL)	12.9±1.9	13.4±1.6	0.054
Platelets* (10^3^/µl)	201.0 (168.0-253.0)	254.0 (214.0-298.3)	<0.001**
Neutrophils* (10^3^/µl)	4.6 (3.4-5.8)	4.3 (3.6-5.3)	0.062**
Lymphocytes* (10^3^/µl)	1.4 (1.0-1.8)	1.7 (1.4-2.3)	<0.001**
Monocytes* (10^3^/µl)	0.5 (0.3-0.6)	0.5 (0.4-0.6)	0,086**
MPV (fL)	9.7±1.1	9.2±0.8	<0.001
MCV (fL)	86.4±6.5	86.7±5.8	0.674
RDW (%)	15.2±2.0	14.6±2.0	0.300
PLR	201.8±153.2	155.1±61.4	0.071
LMR	3.6±2.4	4.3±1.9	0.125
MPVPCR	0.05±0.02	0.04±0.01	<0.001
MPVLR	9.18±6.8	5.6±1.9	<0.001
RDWPCR	0.08±0.06	0.06±0.02	0.013
hs-CRP	14.1±6.0	1.6±1.06	<0.001

* Data are presented as median with interquartile range;

** Mann-Whitney U test.

hs-CRP=high-sensitivity C-reactive protein; LMR=lymphocyte-to-monocyte ratio; MCV=mean corpuscular volume; MPV=mean platelet volume; MPVPCR=mean platelet volume-to-platelet count ratio; MPVLR=mean platelet volume-to-lymphocyte ratio; PLR=platelet-to-lymphocyte ratio; RDW=red cell distribution width; RDWPCR=red cell distribution width-to-platelet count ratio

**Fig. 1:**
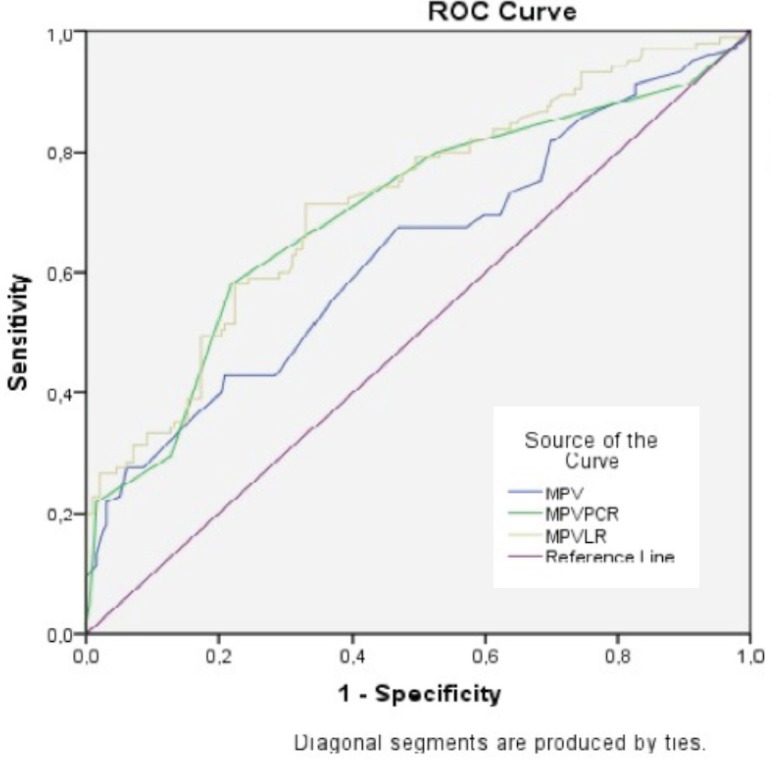
Mean platelet volume (MPV), mean platelet volume-toplatelet count ratio (MPVPCR), mean platelet volume-to-lymphocyte ratio (MPVLR).

**Fig. 2:**
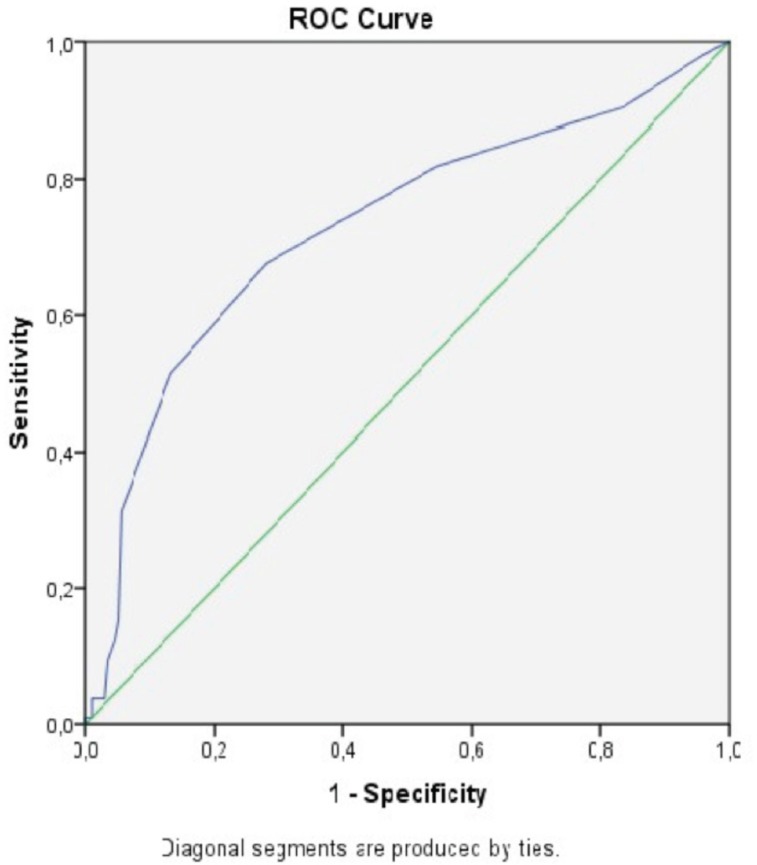
Red cell distribution width platelet count ratio.

In the present study, the optimal cutoff value determined by ROC analysis for MPV was found to be 9.35 [area under the curve (AUC): 0.63, 95% confidence interval (CI): 0.56-0.70, sensitivity: 62%, specificity: 58%], for MPVPCR, this value was 0.04 [AUC: 0.70, 95% CI: 0.63-0.76, sensitivity: 80%, specificity: 48%], for MPVLR, this value was 5.55 [AUC: 0.72, 95% CI: 0.66-0.78, sensitivity: 72%, specificity: 61% (Table 3, Figure 1)] and for RDWPCR, this value was 0.07 [AUC: 0.73, 95% CI: 0.67-0.79, sensitivity: 68%, specificity: 72% (Table 3, Figure 2)].

**Table 3:** Cutoff values, sensitivity and specificity of MPV, MPVLR, MPVPCR and RDWPCR for predicting marker chronic inflammatory of ATAA.

	MPV	MPVLR	MPVPCR	RDWPCR
Cutoff value	9.35	5.55	0.04	0.07
Sensitivity	0.62	0.72	0.80	0.68
Specificity	0.58	0.61	0.48	0.72
AUC (95% CI)	0.631 (0,563-0,700)	0.719 (0.657-0.780)	0.698 (0.633-0.763)	0.729 (0.666-0.792)

ATAA=ascending thoracic aortic aneurysm; AUC=area under the curve; CI=confidence interval; MPV=mean platelet volume; MPVLR=mean platelet volume-to-lymphocyte ratio; MPVPCR=mean platelet volume-to-platelet count ratio; RDWPCR=red cell distribution width-to-platelet count ratio

## DISCUSSION

This study was designed to determine the inflammatory process in patients with ATAA, as the first research in the literature using measurements such as MPV, MPVPCR, MPVLR and RDWPCR.

In previous studies, it was accepted that AA dilation is na important predictor of coronary artery disease, ischemic stroke, acute myocardial infarction and cardiovascular mortality^[11]^. Furthermore, it has been shown in previous studies that AA is also an inflammatory process. For example, in histological examinations of TAA patients, in addition to the high levels of inflammatory cells observed in the adventitia layer, T lymphocytes and macrophages appeared in pathogenesis^[12]^ and there were findings supporting the presence of cytokines^[13]^. However, in accordance with the current research, studies on patients with ATAA and chronic aortic dissection, high levels of hs-CRP and white blood cell levels have been another evidence of the presence of inflammatory status^[14]^. In inflammatory cases, it is known that certain parameters of the complete blood count diverse in terms of number and quality.

Proinflammatory cytokines and acute-phase reactants secreted in inflammatory processes affect megakaryocytopoiesis and decrease platelet volume^[15]^. However, MPV levels indicate platelet activity more accurately than platelet count. MPV levels have been shown to decrease in diseases such as active ankylosing spondylitis, rheumatoid arthritis^[16]^, inflammation period of COPD^[17]^ and systemic lupus erythematosus^[18]^. Similarly, the MPV level was also low in this study (Table 2).

In the literature, MPVPCR has been reported to be high in cases such as sepsis^[19]^, hepatic fibrosis^[20]^, peritonitis and pancreatitis^[21]^, and this situation is reported to be associated with mortality. However, in studies performed, it was shown that MPVPCR value is more determinant than MPV value in predicting complications and cardiac mortality due to non-ST segment elevation myocardial infarction disease^[22]^. Inconsistent with previous studies, the MPVPCR value was found to be high in this study (Table 2).

The high MPVLR values obtained in our study are consistent with the results of a previous study, which indicates that the MPVLR level is an independent indicator for predicting both early and late mortality after STEMI, and in-hospital mortality^[10]^. RDWPCR is another inflammatory index that has been recently introduced and has been examined according to the results of routine blood tests. Previously, the increased RDWPCR value has been reported to be positively correlated with the disease scores of inflammatory factors in systemic lupus erythematosus (SLE)^[23]^. Parallel to these studies, RDWPCR was also high in this study (Table 2).

Our study revealed that the cutoff value of MPVPCR in predicting inflammation in patients with ATAA is comparable to the result of the study by Li et al.^[24]^, in which MPVPCR was shown to be a strong independent predictor for in-hospital complications and mortality in patients with aortic dissection. Furthermore, as a result of the study findings, cutoff values of MPVLR and RDWPCR were similar to each other in prediction inflammation in patients with ATAA (Table 3).

## CONCLUSION

The approach of patients with ATAA should be concerned with monitoring patients after diagnosis, preventing possible complications, controlling blood pressure and any cardiovascular risk factors. Based on the findings of the study, values of MPVPCR, MPVLR and RDWPCR might be proposed as a quick and useful screening tool that can contribute to diagnose and monitor the inflammatory process to help the physician decide whether to request additional imaging studies to confirm or refuse the ATAA diagnosis.

### Limitations of the Study

The most important of the limitations of this study is that, since it is a retrospective study with a relatively small patient group, the laboratory findings of certain clinical and inflammatory markers, such as interleukin-6, TNF-α etc., were not available. Another limitation is that only ATAA data can be obtained by computed tomography and these results cannot be confirmed by other imaging methods, echocardiography, and magnetic resonance. The last constraint was that the patients included in the study could not be followed in terms of possible complications and consequences.

**Table:** 

Authors' roles & responsibilities	
YKT	Substantial contributions to the conception or design of the work; or the acquisition, analysis, or interpretation of data for the work; drafting the work or revising it critically for important intellectual content; final approval of the version to be published
GT	Substantial contributions to the conception or design of the work; or the acquisition, analysis, or interpretation of data for the work; drafting the work or revising it critically for important intellectual content; final approval of the version to be published

## References

[1] Hiratzka LF, Bakris GL, Beckman JA, Bersin RM, Carr VF, Casey DE Jr (2010). 2010 ACCF/AHA/AATS/ACR/ASA/SCA/SCAI/SIR/STS/SVM guidelines for the diagnosis and management of patients with thoracic aortic disease: executive summary: a report of the American college of cardiology foundation/American heart association task force on practice guidelines, American association for thoracic surgery, American college of radiology, American stroke association, society of cardiovascular anesthesiologists, society for cardiovascular angiography and interventions, society of interventional radiology, society of thoracic surgeons, and society for vascular medicine. Anesth Analg.

[2] Von Allmen RS, Anjum A, Powell JT (2013). Incidence of descending aortic pathology and evaluation of the impact of thoracic endovascular aortic repair: a population-based study in England and Wales from 1999 to 2010. Eur J Vasc Endovasc Surg.

[3] Günay S, Güllülü NS (2017). Approach to aortic aneurysms in the elderly. Turk Kardiyol Dern Ars.

[4] Pisano C, Balistreri CR, Ricasoli A, Ruvolo G (2017). Cardiovascular disease in ageing: an overview on thoracic aortic aneurysm as an emerging inflammatory disease. Mediators Inflamm.

[5] Hountis PG, Plestis KA (2009). Strategies in the management of extensive descending and thoracoabdominal aortic aneurysms. Hellenic J Cardiol.

[6] Gonzalez-Hidalgo C, De Haro J, Bleda S, Cañibano C, Michel I, Acin F (2018). Differential mRNA expression of inflammasome genes NLRP1 and NLRP3 in abdominal aneurysmal and occlusive aortic disease. Ther Adv Cardiovasc Dis.

[7] Wang X, Meng H, Xu L, Chen Z, Shi D, Lv D (2015). Mean platelet volume as an inflammatory marker in patients with severe periodontitis. Platelets.

[8] Slavka G, Perkmann T, Haslacher H, Greisenegger S, Marsik C, Wagneret OF (2011). Mean platelet volume may represent a predictive parameter for overall vascular mortality and ischemic heart disease. Arterioscler Thromb Vasc Biol.

[9] Milovanovic Alempijevic T, Stojkovic Lalosevic M, Dumic I, Jocic N, Pavlovic Markovic A, Dragasevic S (2017). Diagnostic accuracy of platelet count and platelet indices in noninvasive assessment of fibrosis in nonalcoholic fatty liver disease patients. Can J Gastroenterol Hepatol.

[10] Hudzik B, Szkodzinski J, Lekston A, Gierlotka M, Polonski L, Gasior M (2016). Mean platelet volume-to-lymphocyte ratio: a novel marker of poor short- and long-term prognosis in patients with diabetes mellitus and acute myocardial infarction. J Diabetes Complications.

[11] Gardin JM, Arnold AM, Polak J, Jackson S, Smith V, Gottdiener J (2006). Usefulness of aortic root dimension in persons &gt; or = 65 years of age in predicting heart failure, stroke, cardiovascular mortality, all-cause mortality and acute myocardial infarction (from the Cardiovascular health study). Am J Cardiol.

[12] Ruvolo G, Pisano C, Candore G, Lio D, Palmeri C, Maresi E (2014). Can the TLR-4-mediated signaling pathway be “a key inflammatory promoter for sporadic TAA”?. Mediators Inflamm.

[13] Jia LX, Zhang WM, Zhang HJ, Li TT, Wang YL, Qin YW (2015). Mechanical stretch-induced endoplasmic reticulum stress, apoptosis and inflammation contribute to thoracic aortic aneurysm and dissection. J Pathol.

[14] Yuan SM, Shi YH, Wang JJ, Lü FQ, Gao S (2011). Elevated plasma D-dimer and hypersensitive C-reactive protein levels may indicate aortic disorders. Rev Bras Cir Cardiovasc.

[15] Bath PM, Butterworth RJ (1996). Platelet size: measurement, physiology and vascular disease. Blood Coagul Fibrinolysis.

[16] Kisacik B, Tufan A, Kalyoncu U, Karadag O, Akdogan A, Ozturk MA (2008). Mean platelet volume (MPV) as an inflammatory marker in ankylosing spondylitis and rheumatoid arthritis. Joint Bone Spine.

[17] Ulasli SS, Ozyurek BA, Yilmaz EB, Ulubay G (2012). Mean platelet volume as an inflammatory marker in acute exacerbation of chronic obstructive pulmonary disease. Pol Arch Med Wewn.

[18] Safak S, Uslu AU, Korkmaz S, Tasliyurt T, Senel S, Akyol L (2014). Association between mean platelet volume levels and inflammation in SLE patients presented with arthritis. Afr Health Sci.

[19] Oh GH, Chung SP, Park YS, Hong JH, Lee HS, Chung HS (2017). Mean platelet volume to platelet count ratio as a promising predictor of early mortality in severe sepsis. Shock.

[20] Iida H, Kaibori M, Matsui K, Ishizaki M, Kon M (2018). Ratio of mean platelet volume to platelet count is a potential surrogate marker predicting liver cirrhosis. World J Hepatol.

[21] Djordjevic D, Rondovic G, Surbatovic M, Stanojevic I, Udovicic I, Andjelic T (2018). Neutrophil-to-lymphocyte ratio, monocyte-to-lymphocyte ratio, platelet-to-lymphocyte ratio, and mean platelet volume-to-platelet count ratio as biomarkers in critically ill and injured patients: which ratio to choose to predict outcome and nature of bacteremia?. Mediators Inflamm.

[22] Sun X, Li H, Zhang Y, He F, Lu C (2019). The prognostic value of mean platelet volume to platelet count ratio in older patients with non-ST elevation acute coronary syndrome receiving primary percutaneous coronary intervention: a retrospective study. Minerva Cardioangiol.

[23] Xie S, Chen X (2018). Red blood cell distribution width-to-platelet ratio as a disease activity-associated factor in systemic lupus erythematosus. Medicine (Baltimore).

[24] Li DZ, Chen QJ, Sun HP, Zeng R, Zeng Z, Gao XM (2016). Mean platelet volume to platelet count ratio predicts in-hospital complications and long-term mortality in type A acute aortic dissection. Blood Coagul Fibrinolysis.

